# Partial CHARGE syndrome with bilateral retinochoroidal colobomas associated with 7q11.23 duplication syndrome: case report

**DOI:** 10.1186/s12886-022-02298-x

**Published:** 2022-03-04

**Authors:** Patrick L. Donabedian, Jessica Y. Walia, Swati Agarwal-Sinha

**Affiliations:** 1 Department of Ophthalmology, University of Florida College of Medicine, Gainesville, FL USA; 2 Department of Ophthalmology, Seattle Children’s Hospital, University of Washington, Seattle, WA USA

**Keywords:** CHARGE syndrome, 7q11.23 duplication, Coloboma, Case report

## Abstract

**Background:**

CHARGE syndrome is a relatively common cause of deafness and blindness resulting from failure to form the primordia of specific organs due to deficient contribution of neural crest cell derivatives. The majority of CHARGE syndrome cases are caused by heterozygous mutations in *CHD7* on chromosome 8q21. Those with CHARGE syndrome without *CHD7* mutation typically do not have an identified genetic defect. 7q11.23 duplication syndrome is associated with mild facial dysmorphism, heart defects, language delay, and autism spectrum disorder. In the current literature, 7q11.23 duplication has not been associated with CHARGE syndrome, retinochoroidal colobomas, or significant ear abnormalities.

**Case presentation:**

We describe a patient with 7q11.23 duplication syndrome and clinical CHARGE syndrome with no variant in CHARGE-associated genes.

**Conclusions:**

This case highlights the still incomplete understanding of the pathogenesis of CHARGE syndrome and raises the possibility of a dose-sensitive effect of genes in the 7q11.23 critical region on neural crest differentiation and fate.

## Background

Colobomas of the iris or choroid are rare malformations of the eye that occur when the embryonic optic fissure at either the anterior or posterior pole of the developing eye fails to close during the fifth week of embryogenesis.^1–3^ Small anterior or peripheral posterior colobomas may be asymptomatic. Large anterior colobomas are typically associated with photophobia, while large or central posterior colobomas lead to visual field defects, increased risk of retinal detachment, and impaired central acuity if the macula or nerve are involved. Severe coloboma phenotypes impair global eye development, leading to microphthalmia or anophthalmia. Both syndromic and isolated colobomas are genetically heterogeneous, and tracing the genes underlying these conditions has added much to our understanding of vertebrate eye development. The genetics of isolated and syndromic ocular coloboma are reviewed elsewhere [1].

CHARGE syndrome is characterized by a pattern of developmental anomalies. The CHARGE acronym stands for coloboma and cranial nerve defects, heart defects, atresia of the choanae, retardation of growth and mental development, genital underdevelopment, and ear abnormalities and sensorineural hearing loss. The criteria used to define CHARGE syndrome have been successively refined to be more specific [2–4]. CHARGE syndrome is typically sporadic, but may be familial and inherited in an autosomal dominant manner with high phenotypic variability, even between monozygotic twins [5]. The majority of cases are caused by heterozygous inactivating mutations in *CHD7*, which codes for the transcription regulator chromodomain helicase DNA-binding protein 7 (CHD7) [6]. Failure of CHD7 to play its normal role in regulating genes relating to differentiation and motility of neural crest derivatives, including the neural crest component of the periocular mesenchyme, is the likely etiology of the abnormalities seen in CHARGE syndrome [7].

7q11.23 duplication syndrome is a rare genetic disorder associated with expressive and receptive language delay, mild facial dysmorphisms, hypotonia, heart defects, and cryptorchidism [8, 9]. Comparison to the epidemiology of the reciprocal 7q11.23 deletion syndrome (Williams-Beuren syndrome) indicate it likely goes frequently undiagnosed, and most cases have been identified by genetic testing of cohorts with autism spectrum disorder [10]. 7q11.23 duplication syndrome has never been reported to be associated with congenital anomalies of the globe, and has no known relationship to CHARGE syndrome.

We report a case of clinically atypical CHARGE syndrome associated with 7q11.23 duplication and with no identified *CHD7* variant in a term baby girl born with small anterior and large posterior retinochoroidal colobomas, hemifacial palsy, atrial septal defect, and external ear abnormalities. We review the clinical features of the case and the results of genetic testing and their relationship to the known pathogenesis of CHARGE syndrome and 7q11.23 duplication.

## Case presentation

**Fig. 1:**
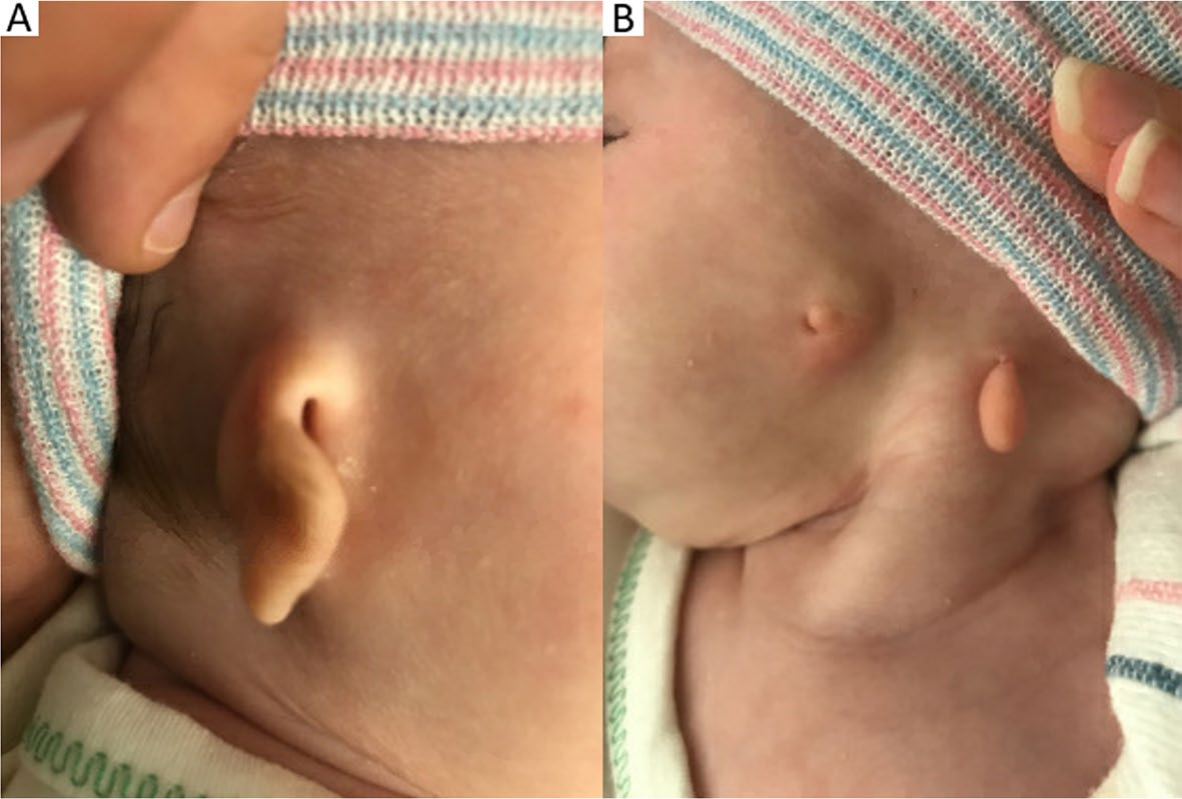
External ears at birth; the left external ear is absent with a postauricular tag and the right ear is hypoplastic

**Fig. 2:**
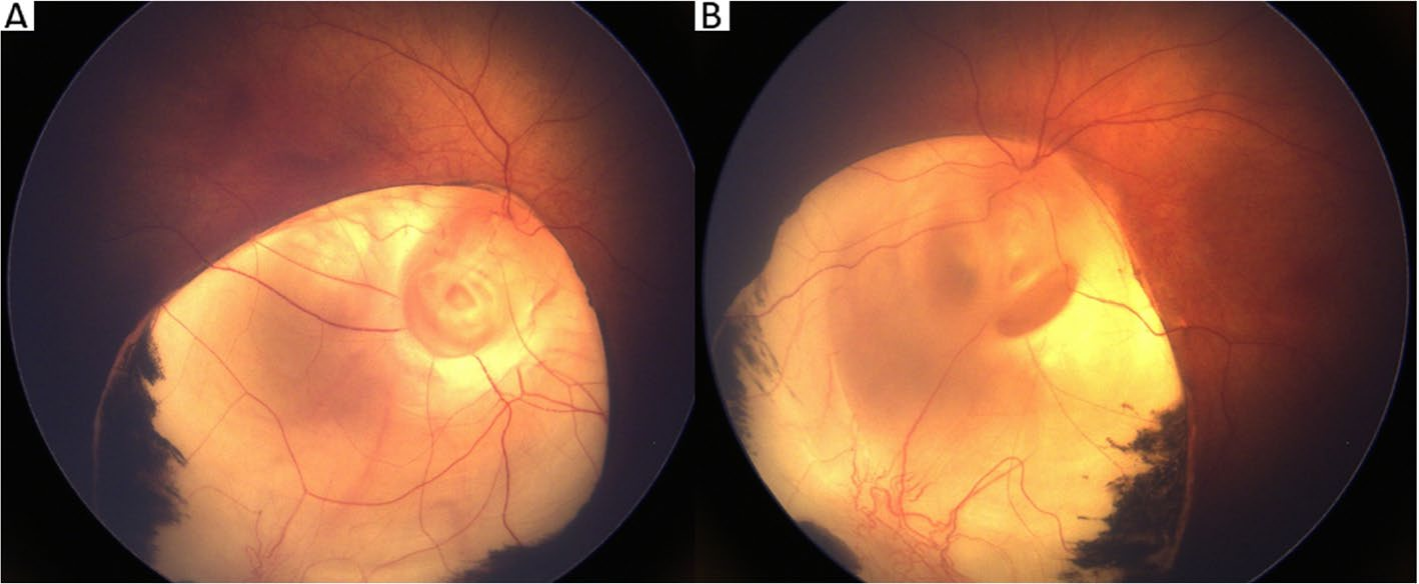
**a** Eyes at 10 months of age; inferior colobomas of the iris in both eyes. **b** Fundus images taken on day of life 5. In both eyes, large inferonasal retinochoroidal colobomas involve the entire optic disc and partial macula

**Fig. 3:**
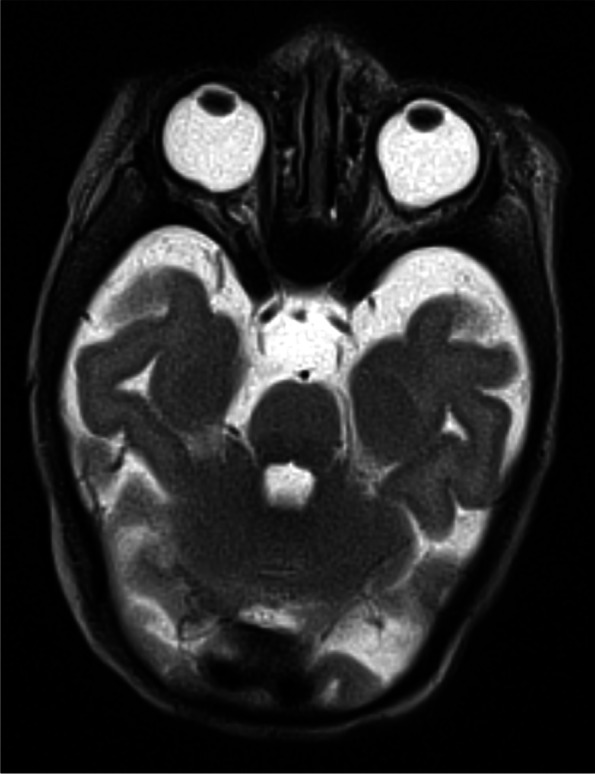
T2-weighted MRI of the brain without contrast taken on day of life 14, showing bilateral outpouchings of the posterior contours of the globes consistent with posterior colobomas

A 4-day-old female, born at 38 weeks gestational age by spontaneous vaginal delivery, was transferred to our academic hospital for care in our neonatal intensive care unit and work-up for multiple facial malformations. She was born to a 23-year-old G1P0 female with maternal family history of Down syndrome and retinitis pigmentosa in, respectively, third- and fourth-degree relatives. On examination after delivery. she was noted to have left-sided anotia with postauricular tag and right-sided microtia (Fig. 1). Full ophthalmic exam at 5-days-old revealed bilateral iris colobomas and bilateral inferonasal retinochoroidal colobomas involving the optic disc and posterior pole, sparing the fovea (Figs. 2a and b). Other systemic findings included bilateral middle ear hypoplasia, atrial septal defect, small perimembranous ventricular septal defect, right hydronephrosis, and right-sided facial hemiparesis. Magnetic resonance imaging of the brain revealed posterior outpouchings of the globes consistent with posterior coloboma (Fig. 3). At 4 and 10 months of age, she was able to fix and follow with central, steady and maintained vision with both eyes. Mild hyperopia (+ 2.00 sphere) was noted on cycloplegic streak retinoscopy. At 15 months, she could walk, look for a hidden object, and stack blocks.

Given findings suggestive of CHARGE syndrome, genetic testing was performed with results summarized (Table 1). Microarray comparative genomic hybridization (SurePrint G3 Human CGH + SNP, Agilent Technologies) and supplemental fluorescence in situ hybridization discovered a duplication of ~ 1.8 Mb of 7q11.23 as well as homozygosity of ~ 8.0 Mb of 6p21.3–22.2. Whole exome sequencing of the patient and both parents (Invitae Whole Exome Boosted Trio, Invitae) discovered no variants in *CHD7*, *SEMA3E*, or other genes related to the phenotype.

**Table 1:** Results of genetic testing

Test	Results	Interpretation
Microarray comparative genomic hybridization and fluorescence in situ hybridization (aCGH & FISH)	Duplication of 1818.7–1858.2 kb of 7q11.23, spanning maximum coordinates of chr7:72,286,211–74,144,421	7q11.23 (Williams-Beuren region) duplication syndrome
	Homozygosity of ~ 8.0 Mb of 6p21.3–22.2, spanning maximum coordinates of chr6:25,859,555–34,007,053	Likely regions identical by descent
Whole exome sequencing to mean depth of 253 × and 99.8% coverage at 20x	Copy number increases in chromosome 7q	Consistent with above
	No variants identified	Normal

## Discussion and conclusions

How should we classify this patient’s findings? Clinical criteria proposed for the diagnosis of CHARGE syndrome are summarized, along with the patient’s characteristics, in Table 2. The original acronym was successively narrowed by Blake in 1998 and Verloes in 2005 to specify CHARGE syndrome and exclude other entities [2, 3]. In 2016, after a decade of research on *CHD7* mutations, Hale proposed widening the criteria to include patients with a partial clinical phenotype and a pathogenic *CHD7* mutation [4]. Despite the widespread availability of genetic testing, CHARGE syndrome remains a clinical diagnosis [11]. In the absence of agreed-upon definitions, we describe this patient as having partial CHARGE syndrome because of the absence of two major criteria such as choanal atresia or cleft plate and pathologic *CHD7* variant.

**Table 2:** Diagnostic criteria for CHARGE syndrome

Pagon (1981) [12]	Blake (1998) [2]	Verloes (2005) [3]	Hale (2015) [4]	This Case Report
**C**oloboma **H**eart malformations **A**tresia of choanae **R**etardation of mental and somatic development **G**enital anomalies **E**ar malformations	**Major criteria** Coloboma Choanal atresia or cleft palate Characteristic ear abnormalities Cranial nerve dysfunction	**Major criteria** Coloboma Choanal atresia Hypoplastic semicircular canals	**Major criteria** Coloboma Choanal atresia or cleft palate Abnormal external, middle or inner ears Pathogenic CHD7 variant	**Major criteria** Coloboma Middle and external ear hypoplasia
	**Minor criteria** Genital hypoplasia Developmental delay Heart or aortic arch malformations Growth hormone deficiency Orofacial cleft Tracheoesophageal fistula Characteristic face	**Minor criteria** Heart or esophagus malformation External or middle ear abnormality Rhombencephalic dysfunction, including sensorineural hearing loss Hypothalamoo-hypophyseal dysfunction (gonadotropin or growth hormone deficiency) Intellectual disability	**Minor criteria** Cranial nerve dysfunction Dysphagia or feeding difficulty Structural brain abnormalities Developmental delay, intellectual disability, or autism	**Minor criteria** Facial hemipalsy, feeding and swallowing difficulty Atrial septal defect and small perimembranous ventricular septal defect
**Inclusion rule** 4 criteria present	**Inclusion rule** 4 major OR 3 major + 3 minor	**Inclusion rule** Typical CHARGE: 3 major OR 2 major + 2 minor Partial CHARGE: 2 major + 1 minor Atypical CHARGE: 2 major + 0 minor OR 1 major + 3 minor	**Inclusion rule** 2 major + any number of minor	

A few years after CHARGE syndrome was first described, the pathogenesis of the syndrome was hypothesized to be due to abnormalities in the migration or maturation of neural crest cell derivatives [13]. In 2004, *CHD7* was identified by sequence analysis of genes in the region of a novel 2.3 Mb microdeletion in 8q12 in two individuals with CHARGE syndrome, and found to be mutated in most CHARGE cases and expressed in the implicated fetal tissues [14]. *CHD7* mutations are detected in 50–90% of cases of CHARGE syndrome, depending on how stringent and which clinical criteria are used; clinical labs report significantly lower diagnostic yield (35%) across all cases of suspected CHARGE syndrome, probably reflecting low, nonspecific thresholds for testing [15]. CHD7 is a chromatin regulator that interacts with other transcription factors to orchestrate transcription of genes essential for certain migratory neural crest (NC) derivatives during embryogenesis [16, 17]. Failure of NC cells to migrate into the periocular mesenchyme is hypothesized to impair reciprocal signaling between ocular and periocular cell populations, leading to complete or partial arrest of optic fissure closure. The other variously present manifestations of CHARGE syndrome represent the failure of specific NC subpopulations to contribute to the formation of specific organ primordia. More than 800 pathogenic *CHD7* mutations have been identified [6, 18], primarily nonsense or frameshift mutations distributed randomly throughout coding regions, as well as some splice site mutations. CHARGE syndrome has also been linked to the *SEMA3E* gene in 7q21.11, once by de novo mutation and once by a novel balanced translocation of chromosomes 2 and 7 [19, 20]. *SEMA3E* codes for a class 3 semaphorin that guides axonal and vascular growth during mouse embryogenesis [21] and is required for migration of cranial neural crest in zebrafish [22]. Pathogenic *CHD7* and *SEMA3E* mutations have also been discovered in cases of Kallmann syndrome (KS) and normosmic hypogonadotropic hypogonadism (nHH), suggesting that some cases of KS/nHH represent a mild CHARGE phenotype where only specialized axonal growth is affected [23, 24]. These investigations link CHARGE syndrome at a genetic or embryologic level to a variety of other syndromes of maldevelopment, including *Sox2* anophthalmia, Alagille, Pallister-Hall and Feingold syndromes, and 22q11.2 deletion (DiGeorge) syndrome.

Other causes for CHARGE syndrome have been proposed, sometimes to explain the many cases without an identifiable genetic variant, as well as the variability in phenotype.

CHARGE syndrome has been reported in a child with a de novo inverted duplication [15] (q22q24.3) [25], in a 6.5 Mb duplication of 2p25 [26], in duplication 8q and deletion 4q from paternal unbalanced translocation t(4;8)(q34;q22.1) [27], in de novo balanced t(6;8)(6p8p;6q8q) [28]; some of these cases predate the availability of clinical sequencing and cannot exclude a co-occuring *CDH7* mutation. De novo mutations of *CHD7* have been found to occur primarily in the paternal germline, suggesting that imprinting may be involved [29]. *CHD7* preferentially localizes to chromatin sites with histone H3, lysine 4 methylation, suggesting that heritable methylation patterns may influence the CHARGE phenotype. Both excess and deficiency of vitamin A have been linked to the anomalies seen in CHARGE syndrome [30, 31]; CHD7 and retinoic acid signaling appear to interact in both inner ear and olfactory bulb development [32, 33].

The clinical features and putative genetic causes of 7q11.23 duplication syndrome bear little relationship to those of CHARGE syndrome, with a distinctive cognitive-behavioral profile (severe language delay with sparing of visuospatial ability, autism spectrum disorder), mild, nonspecific facial dysmorphism and cardiac defects, hypotonia, and normal growth [10]. Single congenital anomalies may be present but are apparently random, and coloboma has not been reported [34]. 7q11.23 duplication syndrome is diagnosed at a much lower rate than the reciprocal 7q11.23 deletion (Williams syndrome), even though from a molecular perspective they should be roughly equal in incidence [35]; a fact attributed to the generally mild and nonspecific phenotype, though a contribution from embryonic lethality cannot be excluded. The roughly 28 genes in the critical region have been studied fairly extensively and include genes with roles in embryonic development, connective tissue, cytoskeleton formation, glucose metabolism, chromatin remodeling, and synapse formation as well as several genes with unknown function [10]. Genotype–phenotype correlations are mostly well-established only in the deletion syndrome; excess dosage of gene products in 7q11.23 duplication syndrome is hypothesized to subtly impair development, primarily of the brain. Symmetrical DNA methylation changes have been observed in patients with 7q11.23 duplication and deletion, suggesting that the function of many genes outside of the critical region may be affected [36].

Many hypotheses would explain the findings in our patient. Coverage of flanking intronic regions by exome sequencing makes a splice site mutation unlikely, but an intronic point mutation or insertion/deletion could introduce a non-canonical splice site or impair splicing regulatory elements, contributing to *CHD7* (or *SEMA3E*) haploinsufficiency [37]. Somatic mosaicism for *CHD7* variants has been reported in CHARGE syndrome and would have gone undetected if present at a low level in peripheral blood. An interacting environmental factor is already likely to contribute to most cases of CHARGE syndrome, given drastic differences in clinical findings even between monozygotic twins. Her ~ 1.8 Mb duplication was at the upper end of the typical range seen in 7q11.23 duplication syndrome, but would not involve any novel genes in the breakpoints. The global hypomethylation associated with 7q11.23 duplication may have altered CHD7’s ability to bind to the appropriate chromatin regions. The 7q11.23 duplication may be entirely unrelated to the CHARGE phenotype—a case of “true, true and unrelated.” Overall, this patient’s presentation, and the variability in CHARGE syndrome, indicates that a seemingly monogenic disorder actually involves multiple insults that interact to produce very different phenotypes, a model that has been proposed for idiopathic hypogonadotropic hypogonadism [38].

Visual prognosis in retinochoroidal colobomas is highly variable. Lack of involvement of the fovea [39, 40], absence of cysts and a structurally normal globe and cornea [41] predict good visual acuity later in life. In our patient, coloboma appears to involve the inferonasal macula but spare the fovea, an encouraging sign. Early correction of refractive error and anisometropia and therapy for amblyopia is as important as in any other child. Additionally, likely due to structural abnormalities within the coloboma or at the margins of normal retina [42, 43],  rhegmatogenous retinal detachments are highly prevalent in colobomatous eyes, affecting 4–40% of cases [44–46]. Regular fundoscopic exam is recommended to detect these vision-threatening complications early. Retrospective studies [45, 47] suggest that prophylactic laser photocoagulation may reduce the risk of retinal detachment and conserve long-term visual acuity in eyes affected by retinochoroidal coloboma, but evidence to guide patient selection and timing is scarce. CHARGE patients in general benefit from a multidisciplinary approach, given their variable developmental delays and sometimes profound deficiencies in sight, hearing and smell [48, 49]. Our patient, currently 17 months old, is doing well at home with her parents and is followed in ophthalmology, otolaryngology, cardiology and pulmonology clinics. She has a bone-anchored hearing aid and can say several words. Her parents are aware of her guarded visual prognosis, despite her currently preserved visual acuity, and are in touch with the Florida School for the Blind.

We report a case of CHARGE syndrome associated with 7q11.23 duplication in a baby girl with small iris colobomas, large posterior retinochoroidal colobomas involving the disk and macula, hemifacial palsy, atrial septal defect, and external ear malformations. Whole exome sequencing revealed no variant in *CHD7*, *SEMA3E*, or any other gene related to the phenotype. While failure to detect pathogenic variants is not uncommon in CHARGE syndrome, reflecting our incomplete understanding, cytogenetic abnormalities are unusual. This case adds to the still-incomplete story of the genetics of CHARGE syndrome and raises the possibility of a role for dosage-sensitive genes in the 7q11.23 critical region in NC specification and fate during embryogenesis.

## Data Availability

"The data that support the findings of this study are not publicly available due to their containing information that could compromise the privacy of patient but are available from the corresponding author (SAS) upon reasonable request".

## Abbreviations

CHARGE: Coloboma, heart defects, atresia of the choanae, retardation of mental and somatic development, genital abnormalities, and ear and hearing problems
NC: Neural crest
KS: Kallmann syndrome
nHH: Normosmic hypogonadotropic hypogonadism
